# A systematic review of tests for lymph node status in primary endometrial cancer

**DOI:** 10.1186/1472-6874-8-8

**Published:** 2008-05-05

**Authors:** Tara J Selman, Christopher H Mann, Javier Zamora, Khalid S Khan

**Affiliations:** 1 Department of Reproductive and Child Health, university of Birmingham, Birmingham Women's Hospital, Birmingham, UK; 2 Clinical Biostatistics Unit, Hospital Ramón y Cajal. CIBER Epidemiologia y Salud Publica (CIBERESP). Madrid, Spain

## Abstract

**Background:**

The lymph node status of a patient is a key determinate in staging, prognosis and adjuvant treatment of endometrial cancer. Despite this, the potential additional morbidity associated with lymphadenectomy makes its role controversial. This study systematically reviews the accuracy literature on sentinel node biopsy; ultra sound scanning, magnetic resonance imaging (MRI) and computer tomography (CT) for determining lymph node status in endometrial cancer.

**Methods:**

Relevant articles were identified form MEDLINE (1966–2006), EMBASE (1980–2006), MEDION, the Cochrane library, hand searching of reference lists from primary articles and reviews, conference abstracts and contact with experts in the field. The review included 18 relevant primary studies (693 women). Data was extracted for study characteristics and quality. Bivariate random-effect model meta-analysis was used to estimate diagnostic accuracy of the various index tests.

**Results:**

MRI (pooled positive LR 26.7, 95% CI 10.6 – 67.6 and negative LR 0.29 95% CI 0.17 – 0.49) and successful sentinel node biopsy (pooled positive LR 18.9 95% CI 6.7 – 53.2 and negative LR 0.22, 95% CI 0.1 – 0.48) were the most accurate tests. CT was not as accurate a test (pooled positive LR 3.8, 95% CI 2.0 – 7.3 and negative LR of 0.62, 95% CI 0.45 – 0.86. There was only one study that reported the use of ultrasound scanning.

**Conclusion:**

MRI and sentinel node biopsy have shown similar diagnostic accuracy in confirming lymph node status among women with primary endometrial cancer than CT scanning, although the comparisons made are indirect and hence subject to bias. MRI should be used in preference, in light of the ASTEC trial, because of its non invasive nature.

## Background

Endometrial cancer is a cancer of the developed world. In Europe it is the most common gynaecological cancer and the fourth most common female cancer after breast, lung and colon cancer [1]. Despite the frequency of this disease the treatment of this cancer, especially in its early stage remains controversial. In 1988 FIGO changed the staging of endometrial cancer to include pelvic and paraaortic lymphadenectomy in acceptance that the lymph node status is one of the most important prognostic factors for a patient [2]. This led to large variations in practice throughout the UK and Europe. A Study of Gynaecological Oncologists in Western Europe revealed only 24.4% performed lymphadenectomy and that despite it's inclusion as part of FIGO staging most reserved it for specific pathological conditions [3].

Advocates for lymphadenectomy demonstrate that it allows precise determination of prognosis, accurate tailoring of adjuvant therapy, and may potentially provide a small survival advantage [4]. Others argue that routine lymphadenectomy is associated with an increased operative time averaging an extra 30 minutes, an increased risk of intraoperative complications and that lymphadenectomy is not necessary in women with good prognostic factors that are at low risk of lymph node involvement. Women with stage 1a-1c disease have less than 0–15% chance of lymph node metastasis.

In light of the controversy surrounding the benefits and risks of lymphadenectomy in patients with endometrial cancer there is increasing interest in minimal and non invasive techniques to determine their lymph node status. Potentially the introduction of a reliable technique could direct the most appropriate patient treatment without the unnecessary risk of lymphadenectomy. As in other cancers studies have investigated the use of imaging techniques and sentinel node biopsy, but the accuracy of these modalities has not been adequately assessed. We systematically reviewed the evidence for the accuracy of minimally invasive and non invasive tests to determine the lymph node status in women with primary endometrial cancer.

## Methods

We used widely recommended methodology in the design of our protocol for the systematic review of the literature [5,6].

### Sources

Our search attempted to capture all the studies that reported the diagnostic accuracy of sentinel node biopsy, positron emission tomography (PET), magnetic resonance imaging (MRI), computer tomography (CT) and ultrasound scanning for the detection of lymphatic spread in primary endometrial cancer. Bibliographic databases MEDLINE (1966–2006), EMBASE, Cochrane Library (issue II, 2006) and MEDION (1980–2006) were searched without language restrictions. The search strategy used relevant medical subheadings (MeSH), text words and word variants for endometrial cancer and combined these with the terms for the index tests and lymphadenopathy (see Additional file 1). Hand searches of reference lists from primary articles and other reviews were carried out to identify manuscripts missed by electronic searching. Experts in the field were contacted for unpublished studies and conference abstracts were reviewed.

### Study selection and data extraction

The selection of studies involved a two-stage process and two reviewers (TJS, CHM). The electronic searches were examined and complete manuscripts of potentially relevant citations retrieved for a final decision on inclusion based on pre-defined selection criteria. Studies were selected if they reported accuracy of the index tests, compared to histological examination of the lymph nodes (reference standard) in women with a primary presentation of endometrial cancer of any histological type or stage and allowed data extraction to create two by two tables. No language restrictions were applied. In cases of duplicate publications the most recent manuscript was selected. Final inclusion or exclusion was decided after examining the complete manuscripts. All were examined in duplicate by the two reviewers with any discrepancies resolved by a third reviewer (KSK).

A piloted data extraction form was used to collect information on study characteristics, quality and accuracy results from each of the selected manuscripts. The study characteristics extracted were the stage of disease, the index test and reference standard methodology and the setting and date of the study. Accuracy data from the studies were reordered in two by two tables. For the purpose of analysis when a manuscript reported the accuracy of more than one index test, the tests were reported on separately. Non diagnostic test results and a failure to perform the test, such as an inability to detect the sentinel node or inadequate histology were excluded from the two by two tables, but their occurrence was recorded, along with the results from the reference standard in each case, if provided.

### Assessment of Study Quality

All of the manuscripts meeting the selection criteria were assessed for their methodological quality, defined as the confidence that the study design, conduct and analysis minimised biases in the estimation of test accuracy. Existing, well developed tools were used to generate items for our assessment of methodological quality [7-9], this process was again carried out in duplicate. For the population, consecutive or random recruitment of eligible women in to the study was considered ideal. Convenience sampling, such as arbitrary recruitment or non-consecutive recruitment was deemed inadequate. Prospective recruitment of patients was considered to be associated with potentially a lesser degree of bias than retrospective recruitment. The description of the population was considered ideal if the study clarified the stage of disease and the body mass index of a patient, which can affect the accuracy of techniques. We recorded the stage of disease in accordance with FIGO classification. The reporting of the index test was considered ideal if the study documented the test in sufficient detail to allow replication by other researchers. It was considered important for the time interval between the index test and the reference standard to be described and an interval of four or less weeks was considered suitable [10]. For the reference standard itself, a description of method of histological verification was important and it was considered preferable for the readers of the reference standard to be blind to the index test results. Information on the number of women recruited into the study and those on whom outcome data were known was sought from the manuscripts to examine partial and differential verification. Verification was considered ideal if all women originally enrolled into the study, without legitimate exclusions were included in the data analysis. We examined if withdraws from the study were explained and if uninterpretable results were reported.

The main strengths and weaknesses in respect of each of the above items for all studies included in the systematic review were tabulated. We did not attempt to collapse our assessment of quality into a score, as suggested methods have little validity and may have a tendency to obscure the strengths and weaknesses of a study rather than clarify them.

### Data synthesis

From the two by two tables, sensitivity (true positive rate) and specificity (true negative rate), along with their exact confidence intervals were computed. These estimates were plotted in a ROC space to evaluate the degree of correlation between these indices. When two by two tables contained zero cells we applied a standard correction of adding 0.5 to all four cells of that table [11].

We anticipated that in common with other diagnostic reviews [10,12,13] there would be heterogeneity of results amongst involved studies. We examined heterogeneity visually using forest plots of sensitivity, specificity and LRs and statistically using Cochran Q [14]. The small number of studies did not allow for detailed exploration of reasons for heterogeneity using meta regression techniques. However studies were instead divided into index test type, which in previous reviews has represented a major source of heterogeneity [16] and difference in accuracy were tested for statistical significance.

We used bivariate random-effect meta-analysis [16] to obtain summary estimates of sensitivity and specificity and other derived measures such as positive and negative likelihood ratios (LRs). LRs allow estimation of the probability of lymphatic spread with a specific test result [17-19]. The bivariate model assumes that logit transformations of sensitivity and specificity are negatively correlated and follow a bivariate normal distribution. This analysis also incorporates the different precision by which sensitivity and specificity have been measured in each study. The model produces random effect estimations for the mean logit sensitivity and specificity with corresponding 95% confidence intervals, it produces also an estimation for the amount of between-study variation for sensitivity and specificity separately, and finally an estimation of the covariance between sensitivity and specificity. Confidence regions in logit-ROC space can be constructed using these estimates. The ellipse in logit-ROC space can be back-transformed to conventional scale, and plotted in ROC space giving a confidence region for the summary operating point.

Meta-DiSc version 1.4 [14] was used for initial analyses and forest plots and the PROC MIXED procedure in SAS version 8.2 for Windows (SAS Institute) was used to fit bivariate models.

## Results

**Figure 1:**
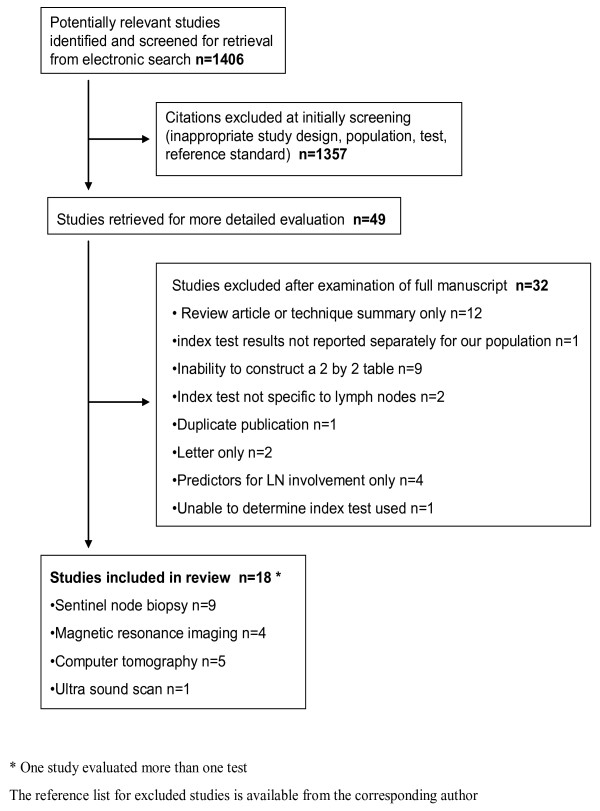
Study selection process for systematic review of literature on accuracy of tests for lymph node metastasis in endometrial cancer.

**Figure 2:**
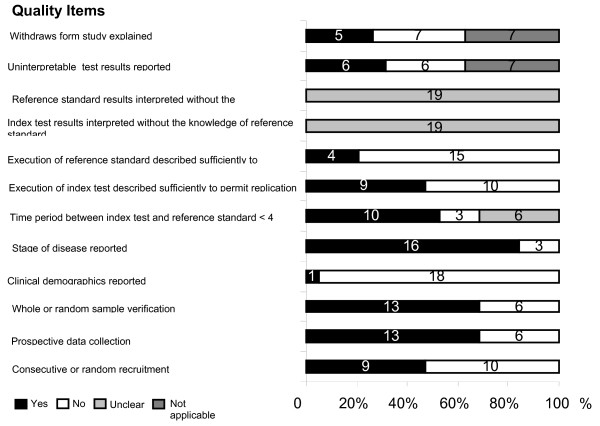
The quality of studies included in systematic review of literature on accuracy of tests for lymph node metastasis in endometrial cancer. Stacked bar chart used. Numbers in bars indicate number of studies.

A total of 18 manuscripts including 693 women with primary endometrial cancer were included in the review [20-37] (Figure 1). There were 19 two by two tables evaluating one of four index tests, there were no studies identified that reported the accuracy of PET. A proportion of the population (106/693, 15%) were included more than once in 2/19 two by two tables. Table 1 and Figure 2 summaries the salient features and quality of each of the studies. It is evident that there was a wide variation and numerous deficiencies in the methodological quality of the included studies.

**Table 1:** Diagnostic accuracy of tests to determine lymph node metatasis in primary endometrial cancer: study characteristics

**Author and Index Test**	**Year**	**Population**	**Setting**	**Index test and failure rate**	**Reference Standard Histological method**
**SN**					
Burke TW	1996	15 women recruited 15 women had index test and reference standard Stage: not stated Pelvic and paraaortic selective lymphadenectomy Open surgery	Hospital – Not stated Country – USA Dates – not stated	SN biopsy using 3 mls blue dye injected into subserosal myometrium 1 women unable to identify SN, positive lymph node status	Histological method not stated
Echt M	1999	8 women recruited 8 women had index test and 7 reference standard Advanced disease prevented lymphadenectomy Stage:IB Pelvic and paraaortic lymphadenectomy Open surgery	Hospital – Alton Ochsner Medical Foundation and University of South Florida Collage of Medicine Dates – 01/01/1993 – 31/03/1995	SN biopsy using 2 mls blue dye injected into uterine funds In all 7 women unable to identify SN, 1 women positive histology	Histological method not stated
Holub Z	2001	8 women recruited 8 women had index test and reference standard Stage:IA-1, IB-5, IC-1, IIIC-1 Pelvic lymphadenectomy Laparoscopic surgery	Hospital – Not stated Country – Czech Republic Dates – 01/200 – 11/2000	SN biopsy using 2 mls blue dye injected into subserosal myometrium 3 women unable to identify SN, all women histology negative	Histological method not stated
Niikura H	2003	28 women recruited 28 women had index test and reference standard Stage: IA-7, IB-11, IIA-2, IIB-1, IIIA-1, IIIC-2 Pelvic and paraaortic lymphadenectomy Open surgery	Hospital – Tohoku University School of Medicine Country – Japan Dates – 06/01 – 01/03	SN biopsy using 70 MBq technetium-99 m colloidal albumin injected hysteroscopically into endomertium 5 women unable to identify a SN, 1 women positive histology	H and E staining and mmunohistochemistry
Pelosi E	2003	16 women recruited 16 women had index test and reference standard Stage: Ib-16 Pelvic lymphadenectomy Laparoscopic surgery	Hospital – Not stated Country – Italy Dates – 02/02 – 04/02	SN biopsy using 37 MBq technetium-99 m colloidal albumin and 4 ml blue dye injected into the cervix 1 women unable to identify SN, negative lymph node status	H and E staining and immunohistochemistry
Raspagliesi F	2003	18 women recruited18 women had index test14 women had reference standard4 excluded, 2 due to disease stageStage IA-4, IB-9, IIIA-1, IIIC-4Pelvic lymphadenectomy in all women, paraaortic only if deemed necessaryOpen surgery	Hospital – Not statedCountry – ItalyDates – Not stated	SN biopsy using 111 MBq technetium-99 m colloidal albumin injected hysteroscopically into sub endomertium	H and E staining
Fersis N	2003	10 Women recruited 10 women had index test and reference standard Stage : Ib pelvic +/- paraaortic lymphadenectomy Open surgery	Hospital – Not stated Country – Germany Dates – Not stated	SN biopsy using 40–100 MBq technetium-99 m colloidal albumin injected hysteroscopically into tumour 3 patients unable to identify SN, patient's lymph node status was negative	Histological method not stated
Holub Z	2004	25 women recruited 25 women had index test and reference standard Stage: not stated Pelvic lymphadenectomy (yes patent had sampling only) Laparoscopic surgery	Hospital – Not stated Country – Czech Republic Dates – 02/00 – 08/03	SN using 5 ml blue dye injected into cervix and uterine fundus 4 patients unable to identify SN, patients lymph node status was negative	Histological method not stated
Lelievre L	2004	12 women recruited 12 women had index test and reference standard Stage: Ib-2, Ic-5, IIa-1, IIb-1, IIIc-3 Pelvic lymphadenectomy Laparoscopic surgery	Hospital – Not stated Country – France Dates 01/02 – 12/02	SN biopsy using 120 MBq technetium-99 m colloidal albumin injected in to the cervix and 2 mls blue dye injected into the cervix 11 patients had SN identified using combined technique, 10 using technetium-99 m alone and 9 using blue dye alone	H and E staining and immunohistochemistry
**CT**					
Balfe DM	1983	61 women recruited 18 women had index test and reference standard 43 women excluded without explanation Stage: not stated Pelvic and paraaortic lymphadenectomy Open surgery	Hospital – Mallinckrodt Institute of Radiology Country – USA Dates – 07/76 – 07/81	CT using EMI CT500S and EMI 7070 3s scanners Lymph nodes > 10 mm abnormal	Histological method not stated
Varpula MJ	1993	47 women recruited47 women had index test43 women had index test and reference standard, 4 women excluded as suitable for dxt onlyStage: I-36, II-7Pelvic and paraaortic unilateral and bilateral clearance and samplingOpen surgery	Hospital – Not statedCountry – FinlandDates – 05/87 – 05/90	CT scan using Siemans Somatom CR/General Electric 9800 scannerslymph nodes > 10 mm abnormal	Histological method not stated
La Fianza A	1997	125 women recruited 125 women index test and reference standard Stage: I-125, II-12, III-8 Pelvic lymphadenectomy Open surgery	Hospital – Not stated Country – Italy Dates – 01/1996 – 09/1993	CT using III generation Somatom 2, Somatome Plus and Siemens scanner	Histological method not stated
Conner JP	2000	702 women were eligible, 210 excluded follow up at another centre, secondary malignancy or no surgery planned 487 women excluded as no CT 75 women had index test 56 women had reference standard, 6 had no lymphadenectomy due to index test results, 13 no explanation Stage : I-350, II-73, III-49, IV-20 Pelvic and paraaortic lymph node sampling Open surgery	Hospital – University of Iowa Hospital and Clinics Country – USA Dates – 1979 – 1993	CT scanner model not stated	Histological method not stated
Zerbe M	2000	54 women recruited 54 women index test 36 women reference standard, no explanation for exclusion Stage I–III Lymph node type not stated Surgery type not stated	Hospital – Baltimore Medical Centre Country – USA Dates – 01/90 – 12/98	CT scanner model not stated No definition for lymph node abnormality	Histological method not stated
**USS**					
Sawicki W	2003	90 women recruited90 women had index test and reference standardStage – not statedlymph node type not statedSurgery type, not stated	Hospital – Not statedCountry – PolandDates – Not stated	USS either abdominal or transvaginal using Siemens Sonoline Versu Prox with a 6.5–7.5 MHZ probe for transvaginal and 3.5 MHZ probe for abdominal definition for lymph node abnormality	Histological method not stated
**MRI**					
Hricak H	1991	20 women recruited 20 women had the index test and reference standard Stage: I-16, II-1, III-3 Pelvic lymph node sampling Open surgery	Hospital – Not stated Country – USA Dates – 01/02/89 – 01/12/89	MRI using 1.5T Sigma and 1.5T Magnetom scanner lymph nodes > 10 mm abnormal	Histological method not stated
Varpula MJ	1993	46 women recruited 46 women had index test 43 women had the index test and reference standard, 3 excluded as suitable for DXT only Stage: I-36, II-7 Pelvic and paraaortic unilateral and bilateral clearance and sampling Open surgery	Hospital – Not stated Country – Finland Dates – 05/87 – 05/90	MRI using < 0.05T Acut scanner Lymph nodes > 10 mm abnormal	Histological method not stated
Taieb S	2002	86 women recruited 86 women had index test and reference standard Stage: Ia-24, Ib-26, Ic-14, IIa-2, Iib-2, IIIc-15, IVa-2, IVb-1 Pelvic and paraaortic lymphadenectomy Open surgery	Hospital – Not stated Country – France Dates – 01/97 – 03/02	MRI scanner type not stated	Histological method not stated
Manfredi R	2004	37 women recruited 37 women had index test 21 women had reference standard, 16 women excluded as no lymph nodes were palpable Stage: Ia-2, Ib-20, Ic-15 Pelvic lymph node sampling in 11, pelvic and paraaortic lymphadenectomy in 10 Open surgery	Hospital – Not stated Country – Italy Dates – 06/97 – 02-01	MRI using 1.5T Echospeed, GE medical System scanner Lymph nodes abnormal > 10 mm	Histological method not stated

SN = sentinel node MRI = magnetic resonance imaging H and E staining = hematoxylin and eosin staining. CT = computer tomography USS = ultrasound scan.

**Figure 3:**
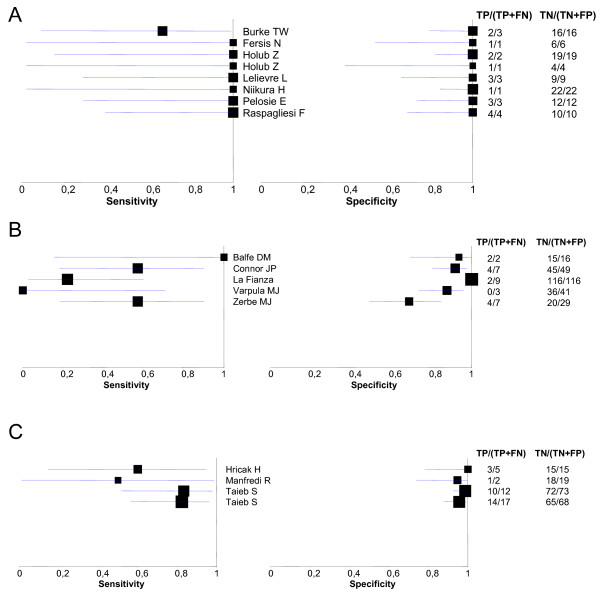
Forrest plots of sensitivity and specificity for the various index tests. A. Sentinel node biopsy B. CT scanning C. MRI.

**Figure 4:**
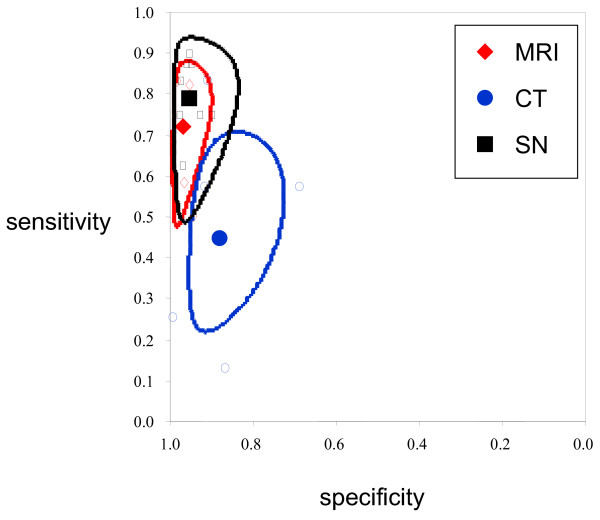
Bivariate summary estimates of sensitivity and specificity for each of the three index tests and the corresponding 95% confidence ellipse around these mean values. Open symbols are individual studies.

Figure 3 show Forrest plots with sensitivities and specificities of individual studies according to index test. Table 2 shows pooled sensitivities and specificities for the various index tests estimated by the bivariate analysis from which we derived other measures such as positive and negative likelihood ratios. Figure 4 shows the summary operating estimates for the various index tests with corresponding confidence ellipses. For each of the index tests variation in sensitivity was much greater than specificity. MRI was the most accurate index test while successful sentinel node had similar results (Table 2). P-values of tests for comparison between the three main diagnostic modalities are shown in Table 2. CT was much less accurate in detection of lymphatic spread (Table 2). There was only one study that reported the accuracy of ultrasound scanning the results of which were positive LR 50.3 and a negative LR 0.67, the presence of only one study makes it difficult to draw a conclusion concerning this technique, other than to note the sensitivity of the test (33%) was poor. The failure rate to detect the sentinel node ranged from 6.6% (1/16 patients) to 100%.

**Table 2:** Summary estimates for sensitivity, specificity and positive and negative likelihood ratios from the bivariate model.

Index test	Mean sensitivity (95%CI)	Mean specificity (95%CI)	Positive likelihood ratio (95% CI)	Negative likelihood ratio (95% CI)
Sentinel Node (SN)	0.79 (0.58 to 0.91)	0.96 (0.89 to 0.99)	18.88 (6.70 to 53.24)	0.22 (0.10 to 0.48)
CT scan	0.45 (0.28 to 0.64)	0.88 (0.78 to 0.94)	3.81 (2.00 to 7.28)	0.62 (0.45 to 0.86)
MRI	0.72 (0.55 to 0.85)	0.97 (0.93 to 0.99)	26.72 (10.56 to 67.64)	0.29 (0.17 to 0.49)

Sensitivity: p-value SN vs CT = 0.018, p-value SN vs MRI = 0.546, p-value CT vs MRI = 0.039.

Specificity: p-value SN vs CT = 0.095, p-value SN vs MRI = 0.546, p-value CT vs MRI = 0.014

## Discussion

Our review showed that MRI and successful sentinel node biopsy (sentinel node biopsy has a variable failure rate) were the most accurate tests for predicting the lymph node status of women with primary endometrial cancer. Other tests were poor in accuracy. These results must be interpreted with caution as the quality of studies available for review was variable, with many of poor methodological quality that may result in the introduction of bias. This review show an urgent need for the further high quality primary studies that include the use of PET scanning as an alternative test which may be beneficial.

This review provides a robust summary of the available evidence to date and an example of the methodology required to perform a review of diagnostic test accuracy. We performed an extensive search for studies and used well developed methods for quality assessment. The deficiencies in quality made explicit by our review should help improve further research in this area [7]. It is imperative that the new STARD and QUADAS guidelines are followed in the undertaking of such studies so that our inference in the future can be based on high quality review, reducing heterogeneity and the risk of bias. Another criticism of our approach might be that in light of the unexplained heterogeneity in the results, meta analysis should perhaps have been avoided. We also accept that we are combing results of tests over a wide time scale, where the accuracy of the technique may have improved. Also that the comparison of tests is an indirect one and hence subject to bias, especially as there is a wide variation in the spectrum of diseases that the different tests are used in.

Our study shows that based on the currently available evidence MRI is the most accurate tool to determine the lymph node status of patients. It has the advantage of also guiding the surgeon as to the depth of myometrial invasion and potential treatment required. Reviews of test accuracy for lymphatic spread in other gynaecological cancers have shown sentinel node biopsy to be the most accurate test, as have studies in other cancers [15]. However this did not appear to be the case for endometrial cancer as MRI was marginally more accurate, although this was not a statistically significant increase in accuracy over sentinel node biopsy. There was a large variation in the ability to detect the sentinel node. Although this usually occurred in a small percentage of patients in the studies, one study was unable to detect the node in any of its patients [21]. This may have been due the different technique used and the reliance on only blue dye to detect the node (Table I).

The debate regarding the necessity of lymphadenectomy in these patients led to the Medical Research Council funded ASTEC (a study in the treatment of endometrial cancer) trial. One of the study's primary aims is to assess the benefit, or otherwise of pelvic lymphadenectomy in patients where disease is thought to be confined to the corpus. Recruitment for this trial had now closed and the preliminary results were presented at European Society of Gynaecological Oncology in 2005. They suggested that there was no benefit for survival or prevention of recurrence in performing lymphadenectomy for early stage endometrial cancer. If this is confirmed in the final publication of the trial then it adds further importance to the use of a non invasive assessment of lymph node status which would allow a decision to be made on the requirement of adjuvant surgery, which in light of this trial will be the only potential benefit of lymphadenectomy.

## Conclusion

Independent of the results of ASTEC there are still benefits in accurately being able to use a non or minimally invasive technique to predict the lymph node status of patients with primary endometrial cancer. This systematic review of the available evidence suggests that MRI is the most accurate method to do this, however one should be cautious in interpreting the results in view of the number and heterogeneity of the studies available and the large confidence intervals of results. Further high quality studies are required to look at the real potential both of this and other imaging modalities such as PET.

## Competing interests

The authors declare that they have no competing interests.

## Authors' contributions

TS participated in the development of the research design and the search strategy essential for systematic review, in the data analysis and interpretation of the data, in writing the first draft and the critical reading there after and approved the final version. CM participated in the development of the research question, in the data extraction and interpretation and the critical reading of the manuscript and approved the final version. JZ participated in the development of the research strategy, in writing a computer program used in the research, in the data analysis and interpretation, in providing essential statistical and methodological advice and in the critically reading of the manuscript and approved the final version. KK participated in the development of the research design and the search strategy essential for systematic review, in the data analysis and interpretation of the data, in surmounting problems that arouse, in providing essential methodological and statistical advice and in the critical reading of the manuscript and approved the final version.

## Pre-publication history

The pre-publication history for this paper can be accessed here:



## Supplementary Material

Additional file 1 Search strategy. The data provided shows the search strategy used in the systematic review of tests for lymph node status in primary endometrial cancer. Click here for file
