# Supplementary material for: A systematic review of tests for lymph node status in primary endometrial cancer
Source: BMC Womens Health. 2008 May 5;8:8. doi: 10.1186/1472-6874-8-8 (PMC2409306; doi:10.1186/1472-6874-8-8)
Supplement: Additional file 1 — Search strategy. The data provided shows the search strategy used in the systematic review of tests for lymph node status in primary endometrial cancer. [file 1472-6874-8-8-S1.doc]

**Search strategy**

Databases search: MEDLINE and EMBASE

Host:Ovid

Date Search run:

Years covered by search: 1950-2007

Search Strategy

1. exp UTERUS/

2. uterus.mp. [mp=title, original title, abstract, name of substance word, subject heading word]

3. 1 or 2

4. exp UTERINE NEOPLASMS/ or exp UTERINE DISEASES/

5. uterine.tw.

6. 3 or 4 or 5

7. exp ENDOMETRIAL NEOPLASMS/

8. endometrial.tw.

9. 6 or 7 or 8

10. exp Neoplasms/

11. exp UTERINE NEOPLASMS/

12. 10 or 11

13. neoplasm$.tw.

14. 12 or 13

15. cancer.mp.

16. cancer.tw.

17. 16 or 15

18. 14 or 17

19. malignan$.mp.

20. malignan$.tw.

21. 19 or 20

22. 18 or 21

23. tumour.mp.

24. tumour.tw.

25. 23 or 24

26. 25 or 22

27. exp CARCINOMA/

28. carcinoma.tw.

29. 27 or 28

30. 29 or 26

31. 30 and 9

32. exp Lymph Nodes/

33. lymph node$.tw.

34. lymph nodes.mp.

35. 32 or 33 or 34

36. exp Lymphatic Diseases/

37. lymphadenopathy.mp.

38. lymphadenopathy.tw.

39. 36 or 37 or 38

40. 35 or 39

41. 40 and 31

42. exp Tomography, X-Ray Computed/

43. computer tomography.mp.

44. computer tomography.tw.

45. ct.mp. [mp=title, original title, abstract, name of substance word, subject heading word]

46. 42 or 43 or 45

47. exp Magnetic Resonance Imaging/

48. magnetic resonance imaging.mp. or Magnetic Resonance Imaging/

49. magnetic resonace imaging.tw.

50. mri.mp. [mp=title, original title, abstract, name of substance word, subject heading word]

51. mri.tw.

52. 47 or 48 or 49 or 50 or 51

53. exp Ultrasonography/ or exp ULTRASOUND, HIGH-INTENSITY FOCUSED, TRANSRECTAL/

54. ultrasound scanning.mp.

55. exp Ultrasonography, Doppler, Duplex/ or ultrasound scan.mp.

56. ultrasound scan.tw.

57. ultrasonography.tw.

58. 53 or 54 or 55 or 56 or 57

59. exp Lymph Node Excision/ or sentinel node.mp.

60. sentinel node biopsy.mp. or exp Sentinel Lymph Node Biopsy/

61. sentinel node.tw.

62. sentinel node biopsy.tw.

63. 59 or 60 or 61 or62.mp. [mp=title, original title, abstract, name of substance word, subject heading word]

64. radionucleotide imaging.mp.

65. radionucleotide imaging.tw.

66. 64 or 65

67. exp Tomography, Emission-Computed/ or postiron emission tomography.mp.

68. positron emission tomography.tw.

69. pet scan.mp. or exp Positron-Emission Tomography/

70. pet scan.tw.

71. 67 or 68 or 69 or 70

72. lymphoscintigraphy.mp.

73. lymphoscintigraphy.tw.

74. 72 or 73

75. 46 or 52 or 58 or 63 or 66 or 71 or 74

76. 75 and 41

Summary: All text words and word variants were used for the population (endometrial cancer lymph nodes) and the interventions (sentinel node biopsy, magnetic resonance imaging, positron emission tomography, computer tomography and ultrasound). During the search we made no restriction on study design due to the limited number of citations and no language restrictions were made.
